# An Algorithm to Select the Optimal Program Based on Rough Sets and Fuzzy Soft Sets

**DOI:** 10.1155/2014/514369

**Published:** 2014-08-28

**Authors:** Liu Wenjun, Li Qingguo

**Affiliations:** ^1^College of Mathematics and Econometrics, Hunan University, Changsha, Hunan 410082, China; ^2^School of Mathematics and Computer Science, Changsha University of Science and Technology, Changsha, Hunan 410076, China

## Abstract

Combining rough sets and fuzzy soft sets, we propose an algorithm to obtain the optimal decision program. In this algorithm, firstly, according to fuzzy soft sets, we build up information systems; secondly, we compute the significance of each parameter according to rough set theory; thirdly, combining subjective bias, we give an algorithm to obtain the comprehensive weight of each parameter; at last, we put forward a method to choose the optimal program. Example shows that the optimal algorithm is effective and rational.

## 1. Introduction

The real world is full of uncertainty, imprecision, and vagueness. Actually most of the concepts we meet in everyday life are vague than precise. So many authors have become interested in modeling vagueness recently. Traditional tools are not always successful to solve these problems. While a wide range of existing theories such as probability theory, fuzzy set theory, rough set theory [1, 2], vague set theory [3], and interval mathematics [4] are well known and often useful mathematical approaches to model vagueness, each of these theories has its inherent difficulties. In 1999, Molodtsov initiated soft set theory as a new mathematical tool for dealing with uncertainties, that is, free from the difficulties affecting existing methods [5]. This theory has proven useful in many different fields such as decision making [6, 7], data analysis [8], and forecasting.

The soft set model can be combined with other mathematical models. Maji et al. presented the concept of fuzzy soft set [9, 10] which is based on a combination of the fuzzy set and soft set models. By combining the interval-valued fuzzy set and soft set, Yang et al. introduced the concept of the interval-valued fuzzy soft set [11]. In [12], the authors applied the notion of soft sets to the theory of BCK/BCI-algebras. Feng et al. established an interesting connection between rough sets and soft sets [13].

Based on [9, 10], and combining rough set theory, in this paper, we propose an algorithm to obtain the optimal decision program.

## 2. Rough Sets and Fuzzy Soft Sets

In this section, we recall the basic notions of rough sets, soft sets, and fuzzy soft sets.

An information system is a pair *S* = (*U*, *A*), where *U* is a nonempty, finite set called the universe and *A* is a nonempty, finite set of primitive attributes. Every primitive attribute *a* ∈ *A* is a total function *a* : *U* → *V*_*a*_, where *V*_*a*_ is the set of values of *a*, called the domain of *a*.

With every subset of attributes *B*⊆*A*, we associate a binary relation ind(*B*), called an indiscernibility relation, and defined thus ind(*B*) = {(*x*, *y*) ∈ *U* ^2^ : for  every  *a* ∈ *B*, *a*(*x*) = *a*(*y*)}, where *a*(*x*) denotes the value of the object *x* with respect to attribute *a*.

Obviously ind(*B*) is an equivalence relation and ind(*B*) = ⋂_*a*∈*B*_  ind(*a*). If (*x*, *y*) ∈ ind(*B*), then *x* and *y* are indiscernible by *B*. The partition generated by ind(*B*) is denoted by *U*/ind(*B*), which is further abbreviated as *U*/*B*.

Let *S* = (*U*, *A*) be an information system and let *B*, *C*⊆*A*; then [2],

1. knowledge *B* depends on knowledge *C* if and only if ind(*C*)⊆ ind(*B*), denoted as *C*⇒*B*;
2. knowledge *B* and *C* are equivalent, denoted as *B*≅*C*, if and only if *C*⇒*B* and *B*⇒*C*;
3. knowledge *B* and *C* are independent, denoted as *B*≇*C*, if and only if neither *C*⇒*B* nor *B*⇒*C* holds.

Obviously *C*≅*B*, if and only if ind(*C*) = ind(*B*).

Let *S* = (*U*, *A*) be an information system and let *R* ∈ *A*; we say that *R* is dispensable in *S* if ind(*A*) = ind(*A* − {*R*}); otherwise *R* is indispensable in information system *S*. The information system *S* = (*U*, *A*) is independent if each *R* ∈ *A* is indispensable; otherwise the information system *S* = (*U*, *A*) is dependent. *B*⊆*A* is a reduction of *A* if *B* is independent and ind(*B*) = ind(*A*). The set of all indispensable relations in *A* will be called the core of *A* and will be denoted as core(*A*).

The following conditions are equivalent [1]:

1. *C*⇒*B*;
2. ind (*C* ∪ *B*) = ind (*C*);
3. Pos_*C*_(*B*) = *U*, where ${\text{Pos}}_{C}(B)={⋃}_{X\in U/B}\underline{C}X$,  $\underline{C}X=\cup \{Y\in U/C|Y\subseteq X\}$;
4. $\underline{C}X=X$ for all *X* ∈ *U*/*B*.

The above properties demonstrate that if *B* depends on *C* then knowledge *B* is superfluous within the information system, in the sense that the knowledge *C* ∪ *B* and *C* provide the same characterization of objects.

Let *U* be an initial universe of objects and *E*_*U*_ (simply denoted by *E*) the set of parameters in relation to objects in *U*. Parameters are often attributes, characteristics, or properties of objects. Let *P*(*U*) denote the power set of *U* and *A* ⊂ *E*. Following [8, 10], the concept of soft sets is defined as follows.

**Definition 1 (see [5]).**

A pair (*F*, *A*) is called a soft set over *U*, where *F* is a mapping given by *F* : *A* → *P*(*U*).

By Definition 1, a soft set (*F*, *A*) over the universe *U* can be regarded as a parameterized family of subsets of the universe *U*, which gives an approximate (soft) description of the objects in *U*. As pointed in [5], for any parameter *a* ∈ *A*, the subset *F*(*a*)⊆*U* may be considered as the set of *a*-approximate elements in the soft set (*F*, *A*). It is worth noting that *F*(*a*) may be arbitrary: some of them may be empty, and some may have nonempty intersection. For illustration, Molodtsov considered several examples in [5]. Similar examples were also discussed in [7, 8].

Maji et al. [9] initiated the study on hybrid structures involving both fuzzy sets and soft sets. They introduced in [9] the notion of fuzzy soft sets, which can be seen as a fuzzy generalization of (crisp) soft sets.

**Definition 2 (see [10]).**

Let *F*(*U*) be the set of all fuzzy subsets in a universe *U*. Let *E* be a set of parameters and *A*⊆*E*. A pair (*F*, *A*) is called a fuzzy soft set over *U*, where *F* is a mapping given by *F* : *A* → *F*(*U*).

In the above definition, fuzzy subsets in the universe *U* are used as substitutes for the crisp subsets of *U*. Hence it is easy to see that every soft set may be considered as a fuzzy soft set. Generally speaking, *F*(*a*) is a fuzzy subset in *U* and it is called the fuzzy approximate value set of the parameter *a*. Following the standard notations, *F*(*a*) can be written as *F*(*a*) = {(*x*, *F*(*a*)(*x*)) : *x* ∈ *U*}.

It is well known that the notion of fuzzy sets provides a convenient tool for representing vague concepts by allowing partial memberships. In the definition of a fuzzy soft set, fuzzy subsets are used as substitutes for the crisp subsets. Hence every soft set may be considered as a fuzzy soft set. In addition, by analogy with soft sets, one easily sees that every fuzzy soft set can be viewed as an information system and be represented by a data table with entries belonging to the unit interval [0,1]. For illustration, we consider the following example.

**Example 3 (see [10]).**

Suppose that there are six houses in the universe *U* = {*h*_1_, *h*_2_, *h*_3_, *h*_4_, *h*_5_, *h*_6_} and the set of parameters is given by *E* = {*a*_1_, *a*_2_, *a*_3_, *a*_4_, *a*_5_, *a*_6_}, where *a*_*i*_  (*i* = 1,2,…, 6) stand for “beautiful,” “modern,” “cheap,” “in good repair,” “wooden," and “in green surroundings,” respectively. Let *A* = {*a*_1_, *a*_2_, *a*_3_} ⊂ *E* be consisting of the parameters that Mr. X is interested in buying a house. This means that out of the available houses in *U* Mr. X wants to buy the house which is qualified with the attributes in *A* to the utmost extent. Now all the available information on houses under consideration can be formulated as a fuzzy soft set *G* = (*F*, *A*) describing “attractiveness of houses” that Mr. X is going to buy.  Table 1 gives the tabular representation of the fuzzy soft set *G* = (*F*, *A*). We can view the fuzzy soft set *G* = (*F*, *A*) as the collection of the following fuzzy approximations:

(1) F(a1)=beautiful  houses={(h1,0.4),(h2,0.6),(h3,0.5),            (h4,0.9),(h5,0.3),(h6,0.6)},F(a2)=modern  houses={(h1,1.0),(h2,0.5),(h3,0.5),            (h4,0.5),(h5,0.7),(h6,0.4)},F(a3)=cheap  houses={(h1,0.5),(h2,0.6),(h3,0.8),           (h4,0.2),(h5,0.9),(h6,0.5)}.

## 3. The Comprehensive Weight of Each Parameter under *λ* Threshold

### 3.1. Ascertain the *λ*-Level

Give a fuzzy soft set (*F*, *A*) over *U*, we use the following algorithm to ascertain the *λ*-level.

*Step  1*. Turn fuzzy soft set (*F*, *A*) into an information system, such as Table 1.

*Step  2*. Build up fuzzy similarity matrix *R*. We use formula *r*_*ij*_ = (1/*m*)∑_*k*=1_^*m*^(1 − |*F*(*a*_*i*_)(*u*_*k*_) − *F*(*a*_*j*_)(*u*_*k*_)|)  (*i*, *i* = 1,2,…, *n*) to compute the similarity degree of objects *u*_*i*_ and *u*_*j*_. Let *R* = (*r*_*ij*_)_*n*×*n*_; obviously, it is a fuzzy similarity matrix. 

*Step  3*. Turn *R* into a fuzzy equivalence matrix R*; that is, we compute the transitive closure of *R*. 

*Step  4*. Choose the optimal classification threshold to clustering.

In an equivalence matrix R*, we sorted the elements *λ*_*i*_ in R* from big to small; that is, 1 > *λ*_1_ > *λ*_2_ > ⋯>*λ*_*k*_ > 0; let *C*_*i*_ = (*λ*_*i*−1_ − *λ*_*i*_)/(*n*_*i*_ − *n*_*i*−1_), where *n*_*i*_ and *n*_*i*−1_ are the number of objects in the *i*th and *i* − 1th clustering, respectively. If *C*_*j*_ = max⁡_*i*_(*C*_*i*_), then we choose *λ*_*j*_ as the best threshold [14].

### 3.2. The Objective Weight of Each Parameter under *λ* Threshold

In the following, combining rough set theory and fuzzy clustering technic, we put forward an algorithm to get objective weight of each parameter from Table 1.

Input a fuzzy soft set (*F*, *A*) over *U*, *U* = {*u*_1_, *u*_2_, …, *u*_*n*_}; *A* = {*a*_1_, *a*_2_,…, *a*_*m*_} are the parameter sets; the processes of parameter weight algorithm are as such.

*Step  1*. Choose the optimal threshold *λ* according to Section 3.1.

*Step  2*. Classify the objects under the optimal threshold *λ*, and we regard them as the classes of *U*/*A*.

*Step  3*. Delete each *a*_*k*_ ∈ *A*  (*k* = 1,2,…, *m*) from *A*; in the same way, we can obtain the classes under *λ*; we regard them as the classes of *U*/(*A* − {*a*_*k*_}) if *U*/(*A* − {*a*_*k*_}) ≠ *U*/*A*; then *a*_*k*_ is indispensable in *A*, and the significance of *a*_*k*_ is

(2) $$\begin{array}{c} \sigma_{a_{k}}=1-\frac{\left|{\text{Pos}}_{A}\left(A-\left\{a_{k}\right\}\right)\cap {\text{Pos}}_{\left(A-\{a_{k}\}\right)}\left(A\right)\right|}{\left|U\right|}. \end{array}$$

*Step  4*. Normalize the significance of each parameter; we get *α* = (*α*_1_, *α*_2_, …, *α*_*m*_), where *α*_*i*_ = *σ*_*λ*_(*a*_*k*_)/(∑_*i*=1_^*m*^ *σ*_*λ*_(*a*_*i*_)).

### 3.3. The Comprehensive Weight of Each Parameter

In the application of actual problems, different customers have different requests; that is, different customers have different subjective weights of parameters. Suppose there are *m* parameters, and *α*_*i*_, *β*_*i*_  (*i* = 1,2,…, *m*) are the subjective and objective weights of the *i*th parameter, respectively. We denote *α* = (*α*_1_, *α*_2_, …, *α*_*m*_); *β* = (*β*_1_, *β*_2_, …, *β*_*m*_), where ∑_*i*=1_^*m*^ *α*_*i*_ = 1, ∑_*i*=1_^*m*^ *β*_*i*_ = 1, and *α*_*i*_, *β*_*i*_ ≥ 0  (*i* = 1,2,…, *m*).

Suppose the comprehensive weight of the *i*th parameter is *w*_*i*_; we denote *W* = (*w*_1_, *w*_2_, …, *w*_*m*_), where ∑_*i*=1_^*m*^ *w*_*i*_ = 1, *w*_*i*_ ≥ 0  (*i* = 1,2,…, *m*). In order to take into account the bias of not only subjective but also objective information, we can build up the following decision model [15]:

(3) min⁡ F(W)=∑j=1m[u(wj−αj)2+(1−u)(wj−βj)2]s.t ∑j=1mwj=1,wj≥0, j=1,2,…,m,

where 0 < *u* < 1 is the bias coefficient; it reflects the bias degree of customers to subjective and objective.

We do a Lagrange function based on the above formula:

(4) L(W,λ)=∑j=1m[u(wj−αj)2+(1−u)(wj−βj)2]+λ(∑j=1mwj−1).

Let

(5) ∂L∂wj=2u(wj−αj)+2(1−u)(wj−βj)+λ=0∂L∂λ=∑j=1mwj−1=0.

Solve the above equations; we can obtain *w*_*j*_ = *uα*_*j*_ + (1 − *u*)*β*_*j*_, *λ* = 0.

## 4. Algorithm for Selection of the Optimal Program

At present there are many methods to handle decision making problems in fuzzy soft sets. In [10] the decision depends on the score *s*_*i*_, where *s*_*i*_ signifies the number of parameters of relatively larger membership value of object *u*_*i*_. In [16] the decision depends on choice value *c*_*i*_, where *c*_*i*_ signifies the sum of the membership values of all parameters of object *u*_*i*_. The decision that results is not always the same according to the two methods. In both of these algorithms, the authors did not think over the significance of each parameter, whereas in the actual problems, different parameters have different significance, and the significance of parameters will affect the result of decision. In this paper, combining rough sets and fuzzy soft sets, we put up with a weighted comprehensive algorithm to choose the optimal program.

1. Input the fuzzy soft sets (*F*, *A*) over *U*.
2. Compute the significance of each parameter *a*_*i*_ ∈ *A*.
3. Compute the score *s*_*i*_ of *u*_*i*_, ∀*i*, where *s*_*i*_ = ∑_*k*=1_^*m*^ *Fa*_*k*_(*u*_*i*_) · *σ*(*a*_*k*_).
4. The decision is *u*_*j*_ if *s*_*j*_ = max⁡_*i*∈{1,2,…,*n*}_ *s*_*i*_.
5. If *k* has more than one value, then any one of *u*_*k*_ may be chosen.

## 5. Example

In the following, we use an example to account for the effectiveness of this optimal decision algorithm. If there are eight invest programs, which denote *U* = {program 1, program 2, program 3, program 4, program 5, program 6, program 7, program 8} = {*u*_1_, *u*_2_, *u*_3_, *u*_4_, *u*_5_, *u*_6_, *u*_7_, *u*_8_}, each program is described by ten factor indexes, denoted by *A* = {*a*_1_(payback period of investment), *a*_2_(estimate of sales), *a*_3_(overall labor productivity), *a*_4_(expected output tax rate), *a*_5_(expected output of per kilowatt), *a*_6_(expected transportation amount per ton kilometer), *a*_7_(expected annual foreign exchange volume), *a*_8_(level of employment), *a*_9_(technical level), and *a*_10_(benefit of bioenvironment)} [14]; according to the experts, we have

(6) F(a1)={(u1,0.5),(u2,0.3),(u3,0.6),(u4,0.5),    (u5,0.4),(u6,0.2),(u7,0.4),(u8,0.5)};F(a2)={(u1,0.8),(u2,0.5),(u3,0.4),(u4,0.4),    (u5,0.6),(u6,0.3),(u7,0.7),(u8,0.6)};F(a3)={(u1,0.4),(u2,0.7),(u3,0.5),(u4,0.3),    (u5,0.4),(u6,0.3),(u7,0.3),(u8,0.7)};F(a4)={(u1,0.8),(u2,0.6),(u3,0.4),(u4,0.8),    (u5,0.3),(u6,0.4),(u7,0.5),(u8,0.3)};F(a5)={(u1,0.7),(u2,0.5),(u3,0.6),(u4,0.4),    (u5,0.4),(u6,0.3),(u7,0.6),(u8,0.3)};F(a6)={(u1,0.6),(u2,0.8),(u3,0.7),(u4,0.2),    (u5,0.4),(u6,0.5),(u7,0.4),(u8,0.5)};F(a7)={(u1,0.3),(u2,0.5),(u3,0.4),(u4,0.4),    (u5,0.5),(u6,0.5),(u7,0.3),(u8,0.6)};F(a8)={(u1,0.6),(u2,0.4),(u3,0.3),(u4,0.5),    (u5,0.4),(u6,0.6),(u7,0.3),(u8,0.5)};F(a9)={(u1,0.5),(u2,0.6),(u3,0.7),(u4,0.3),    (u5,0.3),(u6,0.6),(u7,0.4),(u8,0.6)};F(a10)={(u1,0.3),(u2,0.6),(u3,0.5),(u4,0.5),    (u5,0.4),(u6,0.7),(u7,0.6),(u8,0.4)}.

*Step  1*. Turn fuzzy soft set (*F*, *A*) into an information system (Table 2).

According to the above fuzzy soft set, we can get the following information system.

*Step  2*. Compute the similarity degree of objects *u*_*i*_ and *u*_*j*_ according to *r*_*ij*_ = (1/*m*)∑_*l*=1_^*m*^(1 − |*F*(*a*_*l*_)(*u*_*i*_) − *F*(*a*_*l*_)(*u*_*j*_)|); thus we obtain the similarity degree matrix *R* = (*r*_*ij*_)_*n*×*n*_.

Through computing, we obtain *R* as follows:

(7) $$\begin{array}{c} R=\left(\begin{array}{cccccccc} 1 & 0.57 & 0.61 & 0.61 & 0.61 & 0.56 & 0.61 & 0.52\\ 0.57 & 1 & 0.66 & 0.6 & 0.65 & 0.6 & 0.64 & 0.65\\ 0.61 & 0.66 & 1 & 0.62 & 0.64 & 0.6 & 0.64 & 0.65\\ 0.61 & 0.6 & 0.62 & 1 & 0.69 & 0.59 & 0.69 & 0.69\\ 0.61 & 0.65 & 0.64 & 0.69 & 1 & 0.6 & 0.69 & 0.72\\ 0.56 & 0.6 & 0.6 & 0.59 & 0.6 & 1 & 0.59 & 0.6\\ 0.61 & 0.64 & 0.64 & 0.69 & 0.69 & 0.59 & 1 & 0.69\\ 0.52 & 0.65 & 0.65 & 0.69 & 0.72 & 0.6 & 0.69 & 1 \end{array}\right). \end{array}$$

*Step  3*. Compute the transitive closure of *R* according to [14].

Through computing, we obtain an equivalence relation R* as follows:

(8) $$\begin{array}{c} R^{*}=\left(\begin{array}{cccccccc} 1 & 0.61 & 0.61 & 0.61 & 0.61 & 0.61 & 0.61 & 0.61\\ 0.61 & 1 & 0.66 & 0.65 & 0.65 & 0.6 & 65 & 0.65\\ 0.61 & 0.66 & 1 & 0.65 & 0.65 & 0.6 & 0.65 & 0.65\\ 0.61 & 0.65 & 0.65 & 1 & 0.69 & 0.6 & 0.69 & 0.69\\ 0.61 & 0.65 & 0.65 & 0.69 & 1 & 0.6 & 0.69 & 0.72\\ 0.6 & 0.6 & 0.6 & 0.6 & 0.6 & 1 & 0.6 & 0.6\\ 0.61 & 0.65 & 0.65 & 0.69 & 0.69 & 0.6 & 1 & 0.69\\ 0.61 & 0.65 & 0.65 & 0.69 & 0.72 & 0.6 & 0.69 & 1 \end{array}\right). \end{array}$$

*Step  4*. Ascertain the optimal threshold.

According to the fuzzy equivalence matrix R*, we choose the optimal threshold; since there is no meaning each object is in a class or all the objects are in a class; we do not think over the rate of change at this time; through computing, we have the following rate of change:

(9) C1=1−0.722−0=0.14;C2=0.72−0.694−2=0.015;C3=0.69−0.666−4=0.015;C4=0.66−0.617−6=0.005.

Since *C*_1_ = max⁡{*C*_1_, *C*_2_, *C*_3_, *C*_4_}, so we choose *λ* = 0.72 as the threshold of classification. And the classes are as follows: *U*/*A* = {{*u*_1_}, {*u*_2_}, {*u*_3_}, {*u*_4_}, {*u*_5_, *u*_8_}, {*u*_6_}, and {*u*_7_}}.

*Step  5*. Compute the objective weight of each parameter under *λ* = 0.72.

Delete *a*_1_ from *A*, under the threshold *λ* = 0.72; in the same way, we can get *U*/(*A* − {*a*_1_}) = {{*u*_1_}, {*u*_2_}, {*u*_3_}, {*u*_4_}, {*u*_5_}, {*u*_6_}, {*u*_7_}, and {*u*_8_}} ≠ *U*/*A*; the significance of parameter *a*_1_ is

(10) $$\begin{array}{c} \sigma_{a_{1}}=1-\frac{\left|{\text{Pos}}_{A-\{a_{1}\}}\left(A\right)\cap {\text{Pos}}_{A}\left(A-\left\{a_{1}\right\}\right)\right|}{\left|U\right|}=\frac{1}{4}. \end{array}$$

In the same way, we can get

(11) σa2=14; σa3=0; σa4=38; σa5=14; σa6=14;σa7=38; σa8=0; σa9=0; σa10=14.

Normalize the significance of each parameter; we can get the objective weight of each parameter; through computing, we have *β* = (0.125, 0.125, 0, 0.1875, 0.125, 0.125, 0.1875, 0, 0, and 0.125).

*Step  6*. Compute the comprehensive weight of each parameter.

Suppose that the subjective weight of each parameter is equal; that is, *α* = (0.1, 0.1, 0.1, 0.1, 0.1, 0.1, 0.1, 0.1, 0.1, and 0.1), and the bias coefficient of some customers is *u* = 0.4; according to the formula *w*_*i*_ = *uα*_*i*_ + (1 − *u*)*β*_*i*_, we can get the comprehensive weight of each parameter; through computing, we have *W* = (0.115, 0.115, 0.04, 0.1525, 0.115, 0.115, 0.1525, 0.04, 0.04, and 0.115).

*Step  7*. Choose the optimal program.

According to formula *s*_*i*_ = ∑_*k*=1_^10^ *w*_*k*_ · *F*(*a*_*k*_)(*u*_*i*_), through computing, we have *s*_1_ = 0.5613, *s*_2_ = 0.5463, *s*_3_ = 0.504, *s*_4_ = 0.457, *s*_5_ = 0.419, *s*_6_ = 0.4273, *s*_7_ = 0.4725, and *s*_8_ = 0.4738. Since *s*_1_ = max⁡_*i*=1_^10^{*s*_*i*_}, we choose program 1 as the optimal program.

Since different decision makers have different preferences, that is, different decision makers have different subjective weights, so, they will have different choices according to the above algorithm.

## 6. Conclusion

Combining rough sets with fuzzy soft set theory, in this paper, we give an algorithm to choose the optimal program. During this algorithm, we think over not only the objective weight but also the subjective bias; this optimal program selection algorithm is effective and rational.

## Figures and Tables

**Table 1:** An information system corresponding to (*F*, *A*).

*U* (program)	*a*_1_	*a*_2_	⋯	*a*_*m*_
*u*_1_	*F*(*a*_1_)(*u*_1_)	*F*(*a*_2_)(*u*_1_)	⋯	*F*(*a*_*m*_)(*u*_1_)
*u*_2_	*F*(*a*_1_)(*u*_2_)	*F*(*a*_2_)(*u*_2_)	⋯	*F*(*a*_*m*_)(*u*_2_)
⋮	⋮	⋮	⋮	⋮
*u*_*n*_	*F*(*a*_1_)(*u*_*n*_)	*F*(*a*_2_)(*u*_*n*_)	⋯	*F*(*a*_*m*_)(*u*_*n*_)

**Table 2:** Information system according to fuzzy soft set (*F*, *A*).

*U*	*a*_1_	*a*_2_	*a*_3_	*a*_4_	*a*_5_	*a*_6_	*a*_7_	*a*_8_	*a*_9_	*a*_10_
*u*_1_	0.5	0.8	0.4	0.8	0.7	0.6	0.3	0.6	0.5	0.3
*u*_2_	0.3	0.5	0.7	0.6	0.5	0.8	0.5	0.4	0.6	0.6
*u*_3_	0.6	0.4	0.5	0.4	0.6	0.7	0.4	0.3	0.7	0.5
*u*_4_	0.5	0.4	0.3	0.8	0.4	0.2	0.4	0.5	0.3	0.5
*u*_5_	0.4	0.6	0.4	0.3	0.4	0.4	0.5	0.4	0.3	0.4
*u*_6_	0.2	0.3	0.3	0.4	0.3	0.5	0.5	0.6	0.6	0.7
*u*_7_	0.4	0.7	0.3	0.5	0.6	0.4	0.3	0.3	0.4	0.6
*u*_8_	0.5	0.6	0.7	0.3	0.3	0.5	0.6	0.5	0.6	0.4
